# The Foot-and-Mouth Disease Carrier State Divergence in Cattle

**DOI:** 10.1128/JVI.00388-16

**Published:** 2016-06-24

**Authors:** Carolina Stenfeldt, Michael Eschbaumer, Steven I. Rekant, Juan M. Pacheco, George R. Smoliga, Ethan J. Hartwig, Luis L. Rodriguez, Jonathan Arzt

**Affiliations:** a Plum Island Animal Disease Center, Foreign Animal Disease Research Unit, Agricultural Research Service, United States Department of Agriculture, Greenport, New York, USA; b Oak Ridge Institute for Science and Education, PIADC Research Participation Program, Oak Ridge, Tennessee, USA; University of Iowa

## Abstract

The pathogenesis of persistent foot-and-mouth disease virus (FMDV) infection was investigated in 46 cattle that were either naive or had been vaccinated using a recombinant, adenovirus-vectored vaccine 2 weeks before challenge. The prevalence of FMDV persistence was similar in both groups (62% in vaccinated cattle, 67% in nonvaccinated cattle), despite vaccinated cattle having been protected from clinical disease. Analysis of antemortem infection dynamics demonstrated that the subclinical divergence between FMDV carriers and animals that cleared the infection had occurred by 10 days postinfection (dpi) in vaccinated cattle and by 21 dpi in nonvaccinated animals. The anatomic distribution of virus in subclinically infected, vaccinated cattle was restricted to the pharynx throughout both the early and the persistent phases of infection. In nonvaccinated cattle, systemically disseminated virus was cleared from peripheral sites by 10 dpi, while virus selectively persisted within the nasopharynx of a subset of animals. The quantities of viral RNA shed in oropharyngeal fluid during FMDV persistence were similar in vaccinated and nonvaccinated cattle. FMDV structural and nonstructural proteins were localized to follicle-associated epithelium of the dorsal soft palate and dorsal nasopharynx in persistently infected cattle. Host transcriptome analysis of tissue samples processed by laser capture microdissection indicated suppression of antiviral host factors (interferon regulatory factor 7, CXCL10 [gamma interferon-inducible protein 10], gamma interferon, and lambda interferon) in association with persistent FMDV. In contrast, during the transitional phase of infection, the level of expression of IFN-λ mRNA was higher in follicle-associated epithelium of animals that had cleared the infection. This work provides novel insights into the intricate mechanisms of FMDV persistence and contributes to further understanding of this critical aspect of FMDV pathogenesis.

**IMPORTANCE** The existence of a prolonged, asymptomatic carrier state is a political impediment for control and potential eradication of foot-and-mouth disease (FMD). When FMD outbreaks occur, they are often extinguished by massive depopulation of livestock due to the fear that some animals may have undiagnosed subclinical infection, despite uncertainty over the biological relevance of FMD virus (FMDV) persistence. The work described here elucidates aspects of the FMDV carrier state in cattle which may facilitate identification and/or abrogation of asymptomatic FMDV infection. The divergence between animals that clear infection and those that develop persistent infection was demonstrated to occur earlier than previously established. The host antiviral response in tissues maintaining persistent FMDV was downregulated, whereas upregulation of IFN-λ mRNA was found in the epithelium of cattle that had recently cleared the infection. This suggests that the clearing of FMDV infection is associated with an enhanced mucosal antiviral response, whereas FMDV persistence is associated with suppression of the host antiviral response.

## INTRODUCTION

Foot-and-mouth disease (FMD) is a viral disease caused by the highly contagious FMD virus (FMDV; genus Aphthovirus, family Picornaviridae), which poses a continuous threat to global livestock industries and small-holder farming (1, 2). The acute phase of FMD, which is characterized by fever, inappetence, and lameness in association with characteristic vesicular lesions in the oral cavity and on the feet and udders, can affect a wide range of both domesticated and wild cloven-hoofed animal species. The severity of clinical FMD varies greatly depending on the virus strain and animal species, as well as the previous exposure or vaccination history of the animal (reviewed in references 3 and 4 to 6). For regions in which FMD is endemic (including Africa, Asia, and parts of South America), the disease causes substantial production losses and compromises food security due to decreased milk yields and growth rates. Additionally, the direct costs and logistical requisites of the recurrent vaccination campaigns that are necessary for control and prospective eradication of the disease add considerable burdens to the agricultural systems of many developing countries. For countries that are free of FMD (including countries in Europe and North America and Australia), a potential incursion of the disease would lead to an immediate crisis in the agricultural sector due to drastic restrictions on the transportation, trade, and export of animals and animal products as well as the vast expense of imposed sanitary measures.

Control of FMD is further complicated by the existence of a prolonged, asymptomatic, persistent infection among ruminant species. Persistent FMDV infection, also referred to as the FMDV carrier state, has historically been defined by the presence of infectious virus in oropharyngeal fluids for more than 28 days after virus exposure (7). FMDV persistence occurs in both vaccinated and nonvaccinated animals, irrespective of clinical manifestations of disease, and may persist for multiple years (reviewed in references 5, 8, and [Bibr B9][Bibr B10][Bibr B11]). Pigs, in contrast, although they are highly susceptible to clinical FMD, do not maintain persistent FMDV infection (12).

The FMDV carrier state constitutes an important limitation to international trade in agricultural products and has had a substantial impact on the establishment of control measures in response to outbreaks. However, the clinical and epidemiological relevance of subclinical FMDV persistence is still a subject of controversy (13, 14). Specifically, there is only very limited evidence of FMDV transmission from persistently infected carriers under natural conditions (reviewed in reference 14); furthermore, successful transmission to cattle has been demonstrated to occur from African buffalo, but not from other species, in experimental studies (15, 16). In contrast, experimental studies have failed to replicate within-species transmission from persistently infected to naive cattle (17–20).

Even though viral persistence is a well-recognized aspect of FMDV pathogenesis, the specific mechanisms of the FMDV carrier state remain unknown. Several investigations have localized the site of FMDV persistence to the bovine pharynx (7, 21–24), with more specific localization to epithelial cells of the nasopharyngeal mucosa (25, 26). Interestingly, this specific anatomic region has also been demonstrated to constitute the site of primary FMDV infection following natural and simulated natural virus exposure (27–30). In a study by Juleff et al. (31), the concept of epithelial persistence of FMDV was partially contradicted by the proposal of an alternative mechanism of entrapment of viable viral particles by follicular dendritic cells (FDCs) in lymph nodes draining the oral cavity and pharynx. That study suggested that the FDC-bound virus would subsequently reinfect mucosal epithelial cells upon release from the lymphoid tissue, thereby leading to shedding of infectious virus. However, thus far, the successful recovery of infectious FMDV from lymphoid tissue of persistently infected cattle has not been demonstrated.

The accepted definition of FMDV persistence is based on the recovery of infectious virus from samples of oropharyngeal fluid (OPF) collected using a probang cup (7). Probang samples consist of mucous secretions and epithelial scrapings from the upper gastrointestinal tract and pharynx, thus providing further evidence that the pharyngeal epithelium is a contributing source of persistent virus. Experimental studies have reported a 50 to 100% incidence of persistent FMDV infection in naive or vaccinated cattle exposed to the virus (17, 18, 21, 24, 32). A limited number of investigations have suggested that the incidence of FMDV persistence in vaccinated cattle can be reduced by the use of a 10-fold increase in the antigen payload of conventional (inactivated) vaccines (33).

The work presented here provides novel insights into the mechanisms of FMDV persistence in vaccinated and nonvaccinated cattle exposed to FMDV A_24_ Cruzeiro by simulated natural inoculation. Antemortem infection dynamics with quantitative measurements of viral loads in serum and secretions were analyzed through 5 weeks after virus challenge. Detailed temporoanatomic mapping of virus in tissues obtained from animals euthanized at predetermined time points through the postacute and persistent phases of infection was performed to elucidate the dynamics of virus clearance versus virus persistence in bovine tissues. Further analyses demonstrated significant negative correlations between the levels of expression of antiviral host factors and FMDV genome quantities within distinct microanatomic tissue compartments of the nasopharyngeal mucosa of carrier cattle.

## MATERIALS AND METHODS

### Animal studies.

Animal experiments were performed in biosafety level 3 agriculture facilities at the Plum Island Animal Disease Center (PIADC), Greenport, NY. The experimental procedures were approved by the Institutional Animal Care and Use Committee of PIADC, which functions to ensure compliance with animal welfare regulations (protocol 209-15-R). All animals (approximately 6-month-old Holstein steers or heifers) were obtained from a certified vendor and were allowed 2 weeks of acclimation in the experimental facilities before the start of the studies.

Forty-six cattle (21 nonvaccinated cattle and 25 vaccinated cattle) were used for the studies. Of these, eight nonvaccinated animals and three vaccinated animals were euthanized at predetermined time points during what was defined as the intermediate phase of infection (7 to 14 days postinfection [dpi]), which was at time points earlier than their FMDV persistence status could be determined. Vaccinated and nonvaccinated steers were housed in separate isolation rooms through all experiments. Cattle were sedated by intramuscular injection of xylazine (0.66 mg/kg of body weight) for virus inoculation and clinical examinations. The sedation was reversed by intravenous injection of tolazoline (2.0 mg/kg).

### Vaccination.

Twenty-five cattle were immunized using a recently licensed recombinant FMDV serotype A vaccine (Ad5-FMD-A; USDA product code 1FM.1R0; manufactured by Antelope Valley Bios, Lincoln, NE). This vaccine construct contains the FMDV A_24_ Cruzeiro P1-2A and 3C^pro^ coding regions in a replication-deficient human adenovirus vector (34–36). Vaccination, consisting of intramuscular injection of the licensed product in combination with a commercially available adjuvant (Enabl; product number 7010101; VaxLiant, Lincoln, NE, USA), was performed 14 days before virus challenge.

Fifteen cattle were immunized using the licensed product dose and formulation at 10^7.2^ 50% tissue culture infective doses (TCID_50_) per animal, while 10 cattle received a 10-fold higher vaccine dose (10^8.2^ TCID_50_).

### Challenge virus and inoculation.

A bovine-derived FMDV strain, A_24_ Cruzeiro, was used for direct inoculation of both vaccinated and nonvaccinated steers. All steers were inoculated with 10^5^ 50% bovine tongue infectious doses (BTID_50_) of FMDV A_24_ Cruzeiro using a recently optimized simulated natural system of direct intranasopharyngeal (INP) inoculation (30; J. M. Pacheco and J. Arzt, unpublished data). In brief, 2 ml of inoculum was deposited within the nasopharynx of sedated steers using a flexible plastic catheter. The length of the catheter was adjusted to ensure precise deposition in the nasopharynx on the basis of the findings of previous dissection studies (J. Arzt, unpublished data). Studies in vaccinated and nonvaccinated cohorts were performed in parallel, with the same preparation of inoculum being used for both categories of animals. Adequate deposition of the inoculum was confirmed by detection of high levels of FMDV RNA in oral and nasal swab specimens immediately after inoculation (data not shown).

### Clinical evaluation and antemortem sample collection.

Samples collected on a daily basis consisted of whole blood collected in serum separation tubes, as well as oral and nasal swab samples. The samples were stored on ice and were centrifuged for harvesting of serum, nasal secretions, and saliva immediately after collection. Oropharyngeal fluid samples were collected twice weekly by use of a probang cup (7) starting at 14 dpi for nonvaccinated steers and 7 dpi for vaccinated steers. Probang samples were diluted with an equal volume of minimal essential medium containing 25 mM HEPES and were cannulated for homogenization of the sample. One aliquot intended for virus isolation (VI) was separated and treated with 1,1,2-trichlorotrifluoroethane (TTE) for dissociation of immune complexes prior to freezing (26, 37). All sample aliquots were frozen at −70°C until further processing.

Clinical examinations, which included inspection of oral cavities and feet on sedated cattle, were performed daily for nonvaccinated steers and every other day for vaccinated steers from 0 to 10 dpi. The progression of clinical infection (lesion distribution) was measured by a semiquantitative cumulative scoring system. In brief, any vesicular lesions observed within the oral cavity (dental pad, tongue, gingiva), lips, or nostrils were given 1 point, with additional points being added for vesicles on any of the four feet (1 point per foot), giving a maximum score of 5.

### Postmortem sample collection.

For the objective of monitoring virus clearance from tissues, two nonvaccinated cattle were euthanized by intravenous injection of pentobarbital at each of four time points during the intermediate phase of infection: 7, 8, 10, and 14 dpi (see Table 1). Additionally, four nonvaccinated steers were euthanized at 21 dpi, with the remaining nine steers of this cohort surviving to 35 dpi (see Table 2). Of the 15 cattle vaccinated with the standard dose, 3 animals were euthanized at 8 dpi (see Table 1) and 1 steer was euthanized at 15 dpi for reasons unrelated to the study. Two vaccinated cattle were euthanized at 21 dpi, and the remaining 9 cattle of this group were kept until 35 dpi (see Table 3). The 10 cattle that received the high-dose vaccination were all kept until 35 dpi (see Table 4).

A standardized necropsy procedure with the collection of 20 distinct tissue samples (see Tables 1 to 4) was performed immediately after euthanasia. Each tissue sample was divided into 30-mg aliquots, which were placed in individual tubes before immediately being frozen over liquid nitrogen. An adjacent specimen from each tissue was divided into two or four replicates, embedded in optimal-cutting-temperature medium (Sakura Finetek, Torrance, CA) in cryomolds, and frozen over liquid nitrogen. Tissue samples were kept frozen in the vapor phase over liquid nitrogen and were transferred to the lab within 2 h after collection for storage at −70°C until further processing.

### FMDV RNA detection.

Two aliquots of each tissue sample collected at necropsy were individually thawed and macerated using a TissueLyser bead beater (Qiagen, Valencia, CA). Fifty microliters of tissue macerate was transferred to 96-well plates, and samples were subjected to quantitative real-time PCR (qRT-PCR) analysis following the protocol described below.

**FIG 1:**
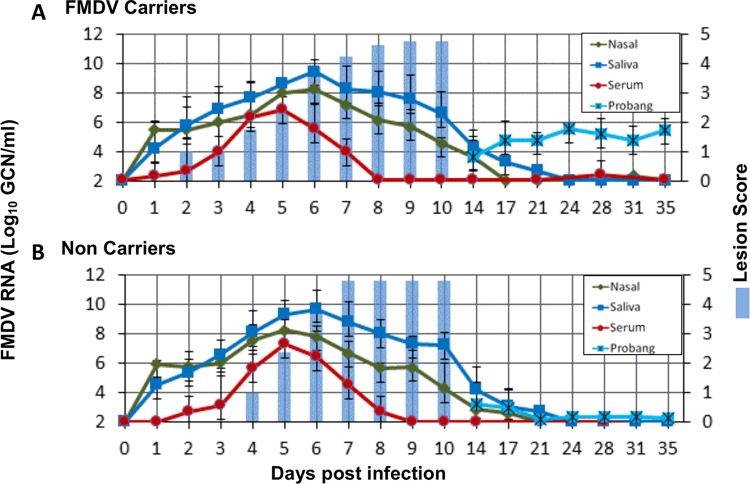
FMDV infection dynamics in nonvaccinated cattle. (A) Results for FMDV carriers (*n =* 8); (B) results for noncarriers (*n =* 5). The amount of FMDV RNA (mean log_10_ GCN per milliliter ± SD) in serum, saliva, and nasal swabs from 0 to 35 dpi and in probang samples from 14 to 35 dpi was measured. The cumulative lesion score, recorded daily from 0 to 10 dpi, provides a semiquantitative measure of the lesion distribution (a lesion score of 5 indicates that vesicular lesions were observed on all four feet and in the mouth). FMDV in probang samples was undetectable from 21 dpi in animals that did not develop persistent infection (B).

**FIG 2:**
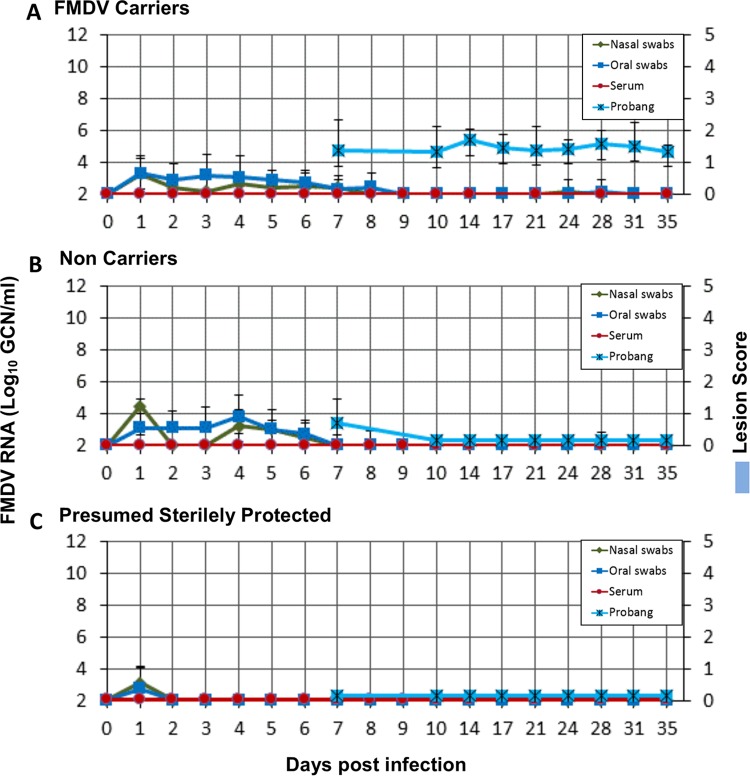
FMDV infection dynamics in vaccinated cattle. (A) Results for FMDV carriers (*n =* 15); (B) results for noncarriers (*n =* 4); (C) results for cattle with presumed sterile protection, as determined by the absence of detection of FMDV RNA in any samples obtained beyond 1 dpi. The amount of FMDV RNA (mean log_10_ GCN per milliliter ± SD) in serum, saliva, and nasal swabs from 0 to 35 dpi and in probang samples from 7 to 35 dpi was measured by qRT-PCR. No clinical lesions were observed in any vaccinated animals at any time. FMDV RNA was undetectable in all probang samples obtained beyond 10 dpi in vaccinated cattle that did not develop persistent infection (B).

Macerated tissue, serum, swab, and probang samples were analyzed using a qRT-PCR targeting the 3D region of the FMDV genome (38) with forward and reverse primers adapted from those of Rasmussen et al. (39) and the chemistry and cycling conditions described previously (12). Cycle threshold (*C_T_*) values were converted to RNA copy numbers per milliliter or milligram on the basis of an external standard of serial 10-fold dilutions of *in vitro*-synthesized FMDV RNA of known concentration. The reported genome copy numbers (GCN) were further adjusted for the average mass of the tissue samples and the specific dilutions used during sample processing. The qRT-PCR results reported in Tables 1 to 4 represent the higher FMDV genome copy number, expressed as the log_10_ GCN per milligram of 2 replicate samples analyzed per tissue per animal. Figures 1 and 2 show the geometric means (40–42) (with geometric standard deviations) of the log_10_ number of FMDV GCN per milliliter for all animals sampled at each time point.

### Virus isolation.

Aliquots of macerated tissue samples and TTE-treated probang samples were cleared of debris and potential bacterial contamination by centrifugation through Spin-X filter columns (pore size, 0.45 μm; Sigma-Aldrich). The cleared samples were subsequently analyzed for infectious FMDV through VI on Lois' and Frances' bovine kidney (LFBK) cells expressing the αvβ6 integrin (43, 44) following a protocol previously described (29). The presence or absence of amplified FMDV was further confirmed by qRT-PCR analysis of the VI cell culture supernatants as previously described (12, 26).

### TTE treatment of tissue samples.

A limited subset of nasopharyngeal tissue samples for which virus isolation was negative, despite the relatively high contents of FMDV RNA and the concurrent detection of infectious virus in probang samples, was subjected to TTE treatment to evaluate if the dissociation of immune complexes could improve the ability to recover infectious virus (37). Similarly, submandibular lymph nodes obtained from both carriers and noncarriers were subjected to the same analysis to evaluate the potential of recovering viable FMDV from lymph nodes during persistent FMDV infection. TTE treatment of tissues was performed as previously described (12). In brief, previously untouched aliquots of the selected samples were homogenized in 1 ml of tissue culture medium. Equal volumes of TTE were added to the tissue homogenates, and the samples were thoroughly mixed through a 2-min cycle in the TissueLyser bead beater. Samples were immediately set on ice and thereafter separated by centrifugation. The clear supernatant was removed from the viscous bottom layer. A total of 250 μl of the supernatant was used for virus isolation following the procedure described above. Tissues collected from cattle euthanized during acute infection (FMDV RNA and VI positive) and corresponding pharyngeal tissue samples from noncarriers (VI negative) from the current study were included as experimental controls.

### Quantification of anti-FMDV IgA in saliva samples.

FMDV-specific IgA in saliva was measured by an adapted immunoassay previously optimized for porcine samples (45). In brief, the IgA response was determined by a sandwich enzyme-linked immunosorbent assay based on the capture of FMDV antigen by use of a rabbit anti-FMDV A_24_ Cruzeiro antiserum (Institute for Animal Health, Pirbright, United Kingdom) and subsequent detection by a sheep anti-bovine IgA antibody directly conjugated to horseradish peroxidase (product number A10-121P; Bethyl Laboratories Inc., Montgomery, TX). The rabbit antiserum was diluted to 1:2,000 in carbonate-bicarbonate buffer (pH 9.6) and adsorbed on Immulon 2 HB microtiter plates (Dynatech Corp., Chantilly, VA) at 100 μl/well. After 4 washes with phosphate-buffered saline (PBS) containing 0.05% Tween 20 (PBS-T), the plates were blocked for 1 h at 37°C with blocking buffer (10% normal horse serum [Sigma-Aldrich, St. Louis, MO] in PBS-T) under agitation. Preparations of inactivated FMDV from infected BHK-21 cells were used as the solid-phase antigen. For each sample well, there was a corresponding negative-control well in which the captured antigen was replaced by similarly prepared uninfected BHK-21 cells. Saliva samples were diluted 1:100 before testing. A positive-control sample consisting of saliva from a persistently infected bovine with known high anti-FMDV IgA contents was included on each plate. Wells were read for the optical density (OD) at 630 nm. Results were calculated as the OD in the positive antigen well minus the OD in the negative antigen well and are reported as the fraction of the vale for the positive control (FPC).

### Laser capture microdissection.

Pharyngeal mucosal tissue samples selected on the basis of prescreening by qRT-PCR and VI were processed using laser capture microdissection (LCM) to determine the microanatomic distribution of FMDV RNA as previously described (30). In brief, four samples representing distinct microanatomic structures (nonlymphoid epithelium [Epith], lymphoid follicle-associated epithelium [FAE], mucosa-associated lymphoid follicles [LF], and submucosa [SM]) were dissected from 10-μm cryosections using an Arcturus XT LCM system. Dissected samples of 150,000 μm^2^ in surface area were captured onto individual CapSure Macro LCM caps (catalog number LCM0211; Life Technologies), which were immediately mounted onto microtubes containing 50 μl of RNA extraction buffer (PicoPure). RNA extraction was performed using a PicoPure RNA isolation kit (catalog number KIT0202; Life Technologies) with a final elution volume of 17 μl.

### Quantification of FMDV RNA in LCM samples.

The FMDV RNA contents in distinct microanatomic compartments were measured using the qRT-PCR protocol described above, accounting for the specific elution and dilution volumes used throughout the procedure. The weight of the sample input in the qRT-PCR assay was estimated on the basis of sample volume (150,000-μm^2^ dissected area × 10-μm section thickness) and an assumed soft tissue density of 1.0 g/cm^3^. The total RNA quantity was determined using a NanoDrop 1000 spectrophotometer (Thermo Scientific), with RNA purity being assessed by measurement of the *A*_260_/*A*_280_ ratio. Glyceraldehyde-3-phosphate dehydrogenase (GAPDH) mRNA levels were quantitated in each sample to assess the consistency of the results and the applicability of the PCR across samples.

### Quantification of host cytokine mRNA expression in LCM samples.

The relative levels of the mRNA of lambda interferon (IFN-λ), IFN-γ, interferon regulatory factor 7 (IRF-7), CXCL10 (IFN-γ-inducible protein 10 [IP-10]), and interleukin-10 (IL-10) were measured in total RNA extracted from the microdissected tissue samples described above. The assay was performed using previously described primers and probes (46) and the gene for GAPDH as the internal reference. Extracted RNA was diluted 5-fold before qRT-PCR analysis was performed using the chemistry and cycling conditions previously described (30). Baseline mRNA expression levels for each cytokine within each distinct tissue compartment were established through a similar analysis of samples of 4 distinct tissues from each of 3 uninfected control steers, generating 12 baseline measurements for each microanatomic compartment (4 tissues × 3 animals). The relative level of expression of each host gene was calculated using the ΔΔ*C_T_* method (47).

### Immunomicroscopy.

After screening of tissue samples for FMDV RNA and infectious virus by qRT-PCR and VI, respectively, detection of antigen in cryosections by immunohistochemistry (IHC) and multichannel immunofluorescence (MIF) was performed as previously described (27, 48). Slides were examined with a wide-field epifluorescence microscope, and images were captured with a cooled, monochromatic digital camera. Images of individual detection channels were adjusted for contrast and brightness and merged in commercially available software (Adobe Photoshop CS2). Additional negative-control tissue sections were prepared from corresponding tissues derived from noninfected cattle. The FMDV nonstructural (3D) protein was detected using the mouse monoclonal antibody F19-6 (49), and the FMDV structural (VP1) protein was detected using mouse monoclonal antibody 6HC4 (50). MIF experiments included labeling of phenotypic cell markers using the following antibodies: rabbit anti-cytokeratin (catalog number 180059; Life Technologies), mouse anti-sheep major histocompatibility complex class II (MHC-II; product code MCA2228; Serotec), and mouse anti-bovine CD11c (catalog number BOV2026; Washington State University).

### Statistical analyses. (i) Statistical analysis of virus shedding.

To compare the total amount of FMDV RNA shed by each animal in nasal, oral, and oropharyngeal (probang) fluids over the course of the study, areas under the curve (AUC) were calculated using the trapezoidal method (51, 52). The mean of the individual AUC values for each group and its 95% confidence interval (CI) were used for between-group comparisons. The difference between the group means was further evaluated with unpaired *t* tests for an alternative hypothesis of no difference. A *P* value of less than 0.05 was considered significant.

### (ii) Statistical analysis of cytokine mRNA expression in LCM samples.

LCM qRT-PCR data were analyzed using a mixed-effects model for each log-transformed dependent variable (DV) (fold change in IFN-γ, IFN-λ, IL-10, CXCL10, and IRF-7 mRNA levels or the FMDV GCN per milligram) using an approach similar to that described previously (53).

Candidate models were constructed with vaccination status, persistence status, and microanatomical region (Epith, FAE, LF, SM) as categorical predictors and the FMDV RNA load as a covariate (for cytokine DVs) and included possible interactions between the predictors. Animals and macroanatomical tissue identifications (dorsal soft palate or dorsal nasopharynx) were included as random effects. The mixed-effects models were compared on the basis of the second-order Akaike information criterion. The best-fitting model for each dependent variable was selected by stepwise elimination of nonsignificant variables.

To test associations between log-transformed continuous variables, Pearson's product moment correlation coefficient (*r*) was calculated and evaluated against a *t* distribution with *n* − 2 degrees of freedom. For each host gene, a linear model was fitted by robust regression with the FMDV RNA load as the single explanatory variable; only the estimated slope β of the regression line (with the 95% confidence interval) is reported. The 95% confidence interval for the estimate was calculated using its standard error and the 97.5% point of a *t* distribution with *n* − 2 degrees of freedom.

In order to more precisely investigate processes potentially involved in the clearance of virus from infected tissues, a similar analysis was performed by including only animals that were euthanized during what was defined as the transitional phase of infection, corresponding to the determined time frame of virus clearance, which was 10 to 21 dpi for nonvaccinated cattle.

An additional semiquantitative analysis was performed in which cytokine mRNA measurements in LCM samples were assigned to four states, separating observations with a greater than 10-fold up- or downregulation (i.e., log_2_ fold changes of >+3.32 or <−3.32, respectively) from observations with less up- or downregulation (log_2_ fold changes of <+3.32 but >0 or >−3.32 but <0, respectively). Carriers and noncarriers were analyzed separately by including only LCM samples with measurable quantities of FMDV RNA from the carrier group. For each cytokine and microanatomical region, the number of observations in each state was divided by the total number of observations to establish the prevalence of any given event (corresponding to the relative up- or downregulation of cytokine mRNA expression). These prevalence proportions were compared between carriers and noncarriers, and the results for the group with the higher relative frequency are shown in Fig. 8.

Data analysis was performed using the R statistical environment with the reshape2, lme4, AICcmodavg, car, phia, lsmeans, and ggplot2 packages. In all analyses, *P* values of <0.05 were considered significant. Where appropriate, *P* values were adjusted using Holm's method (54).

## RESULTS

### Clinical infection.

All of the 21 nonvaccinated cattle included in the study developed moderate to severe clinical FMD after virus exposure. Characteristic vesicular lesions within and around the oral cavity, on the nares and lips, in interdigital clefts, and on coronary bands appeared at 2 to 5 dpi (Fig. 1) and were accompanied by a transient increase in rectal temperature (not shown). The distribution and severity of lesions progressed rapidly over 2 to 4 days after their initial appearance before they gradually declined and resolved within approximately 10 days. Oral lesions consisted of epithelial blanching with subsequent vesiculation and erosions on the dental pad and dorsal surface of the tongue. Most cattle also developed erosions in and around nostrils and on the nasal planum after rupture and sloughing of the vesicle epithelium. Foot lesions included blanched epithelium and vesicles within the interdigital cleft, with more profound vesiculation affecting coronary bands and heel bulbs, and were often accompanied by marked lameness of the affected limbs. Only 3 out of the 21 nonvaccinated animals did not reach full cumulative lesion scores (i.e., a score of 5, indicating lesions on all four feet as well as within the oral cavity or on the muzzle).

There were no vesicular lesions or other signs of clinical FMD after virus exposure of the 25 vaccinated cattle that were included in the study (Fig. 2).

### Antemortem infection dynamics. (i) Nonvaccinated cattle.

Abundant FMDV RNA was detected in both nasal swabs and saliva following intranasopharyngeal (INP) inoculation, confirming the appropriate deposition of the inoculum (not shown). This residual inoculum was cleared within 1 to 2 days with a subsequent consistent increase in nasal and oral shedding of virus from approximately 2 dpi until mean peak levels were reached at 5 and 6 dpi, respectively (8.01 log_10_ GCN/ml for nasal swabs and 9.12 log_10_ GCN/ml for saliva; Fig. 1). The dynamics of nasal and oral detection of virus were largely similar, although viral RNA levels in nasal swabs were marginally higher than those in saliva through the first 24 h after inoculation and slightly lower than those in saliva through the remainder of the clinical phase of infection. Detection of FMDV RNA in nasal swabs and saliva decreased to below the limit of detection (1.57 log_10_ GCN/ml) by 14 to 21 dpi, regardless of whether the animals developed persistent infection or not (Fig. 1). Viremia (defined by the detection of viral RNA in serum) was first detected between 1 and 5 dpi (mean ± standard deviation [SD], 2.96 ± 1.10 dpi) and lasted for 4 to 6 days before it declined to levels below the assay limit of detection (Fig. 1). Peak viremia occurred at between 3 and 6 dpi and reached a mean value of 7.56 log_10_ GCN/ml.

### (ii) Vaccinated cattle.

FMDV infection dynamics were similar in cattle immunized with the standard or a 10-fold vaccine dose, and are therefore described together. FMDV RNA was detected postinoculation in nasal swabs and saliva from all vaccinated animals at levels comparable to those in naive animals (not shown). In the majority of animals, low levels of FMDV RNA were present in nasal swabs and saliva up to 8 dpi, with FMDV RNA being more consistently detected in saliva (maximum level of detection, ≤5 log_10_ GCN/ml; Fig. 2A and B). No viral RNA was subsequently detectable in nasal swabs or saliva, and viral RNA was not detected in the serum of any of the vaccinated cattle at any time (Fig. 2).

Within the group of vaccinated cattle, three steers were distinct in that no FMDV RNA was detected in any sample obtained later than 1 dpi (interpreted as a residual inoculum; Fig. 2C). Additionally, in contrast to all other animals included, no FMDV RNA or infectious virus was detected in tissues from these three individuals (described in detail below). On the basis of these findings, these three animals were treated as a separate group defined to be “presumed sterilely protected.”

### FMDV carrier versus noncarrier divergence in nonvaccinated animals.

On the basis of detection of infectious FMDV in oropharyngeal fluid (probang) samples which were collected twice weekly from 14 to 35 dpi, nonvaccinated cattle diverged into distinct cohorts representing FMDV carriers and noncarriers by 21 dpi (Fig. 1). This determination was based upon the finding that in the subset of animals identified to be persistently infected carriers, probang samples were consistently positive for viral RNA and infectious virus throughout the sampling period, with average values reaching 5.0 log_10_ GCN/ml (Fig. 1A). In contrast, in the noncarriers, viral shedding in probang samples became undetectable at between 14 and 21 dpi (Fig. 1B). Thus, there was no change in FMDV persistence status in any nonvaccinated animal beyond 21 dpi. On the basis of these findings, it was concluded that animals could be categorized as either persistently infected FMDV carriers or noncarriers by 21 dpi. Using this definition, 8 out of 13 nonvaccinated cattle (62%) that survived to 21 dpi or longer were determined to be persistently infected carriers, whereas 5 animals (39%) successfully cleared the infection.

### FMDV carrier-noncarrier divergence in vaccinated animals.

Probang samples were collected twice weekly from vaccinated animals from 7 to 35 dpi. Similar to the nonvaccinated group, there was a clear divergence of the animals into two groups with distinct patterns of FMDV shedding in probang samples. However, the carrier versus noncarrier divergence occurred significantly earlier in the vaccinated animals than in the nonvaccinated group (*P* = 0.005). In cattle that developed persistent infection, there was consistent detection of FMDV RNA and infectious virus in probang samples throughout the sampling period (average peak level, 5.11 log_10_ GCN/ml; Fig. 2A). Among all vaccinated cattle that did not develop persistent infection, there was only one qRT-PCR/VI-positive probang sample, which was collected at 7 dpi. Subsequent to 7 dpi, there was no detection of viral RNA or infectious virus in probang samples from any vaccinated noncarrier animal (Fig. 2B). Twelve animals vaccinated with the standard vaccine dose were kept alive beyond the established threshold of divergence of 10 dpi. When the three animals with no sign of infection (the presumed sterilely protected animals) were excluded, seven out of nine cattle in this group (78%) were determined to be persistently infected carriers, while two (22%) had cleared the infection. Among the 10 cattle receiving the 10-fold higher vaccine dose, all of which were kept until 35 dpi, 8 animals (80%) were carriers and 2 (20%) were noncarriers. The combined prevalence of FMDV persistence following confirmed subclinical infection of vaccinated cattle was thus 79% (this value excludes the presumed sterilely protected animals), whereas it was 67% when the total numbers of vaccinated cattle were included in the calculations (this value includes the presumed sterilely protected animals).

### Quantitative comparison of virus shedding between different cohorts of animals.

**FIG 3:**
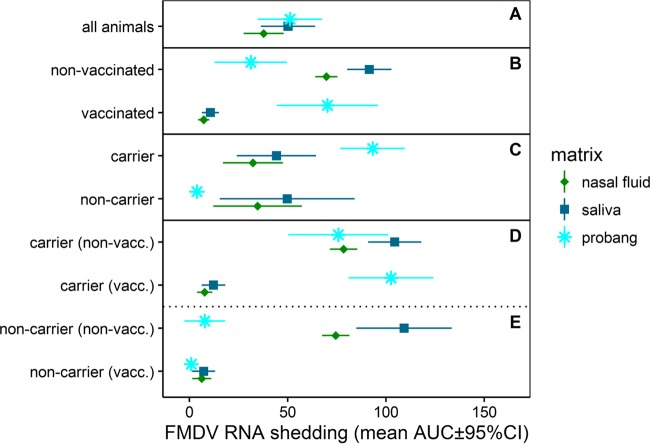
FMDV RNA quantities shed in saliva, nasal fluid, and probang samples stratified by animal cohorts. AUCs are based on the quantities of FMDV RNA shed in different sample types. The data represent the mean of the individual AUC values within each group and its 95% CI. (A) There were no differences in FMDV RNA quantities between the three different sample types when the data for all animal cohorts were combined. (B) Vaccinated cattle shed significantly smaller quantities of FMDV RNA in nasal fluid and saliva. However, virus shedding in probang samples was higher in vaccinated cattle, but the difference was not statistically significant. (C) Persistently infected FMDV carriers shed significantly more viral RNA in probang samples than noncarriers, but there were no differences in the amounts of FMDV RNA shed in saliva and nasal fluid between carriers and noncarriers. (D and E) Four-way stratification of data illustrating a reduction in virus shedding in nasal fluid and saliva associated with vaccination and higher quantities of FMDV RNA in probang samples from FMDV carriers, regardless of vaccination status. Data are for 14 vaccinated carriers [carrier (vacc.)], 8 nonvaccinated carriers [carrier (non-vacc.)], 4 vaccinated noncarriers [non-carrier (vacc.)], and 5 nonvaccinated noncarriers [non-carrier (non-vacc.)].

To compare the amounts of FMDV RNA shed in nasal, oral, and oropharyngeal fluid (probang) samples over the course of the study, areas under the curve (AUC) were calculated using the trapezoidal method (51, 52). Vaccinated cattle shed significantly smaller quantities of viral RNA in nasal fluids and saliva than nonvaccinated cattle (mean AUC ± 95% CI for vaccinated and nonvaccinated cattle, 7.2 ± 2.8 and 69.7 ± 5.6, respectively, for nasal fluids and 10.7 ± 4.4 and 91.6 ± 11.3, respectively, for saliva; *P* < 0.001 for both comparisons; Fig. 3). There was no difference in the quantities of virus shed in nasal fluids and saliva between FMDV carriers and noncarriers. In contrast to this, vaccination did not reduce virus shedding in probang samples (Fig. 3).

### Detection of anti-FMDV IgA in saliva of nonvaccinated and vaccinated cattle.

**FIG 4:**
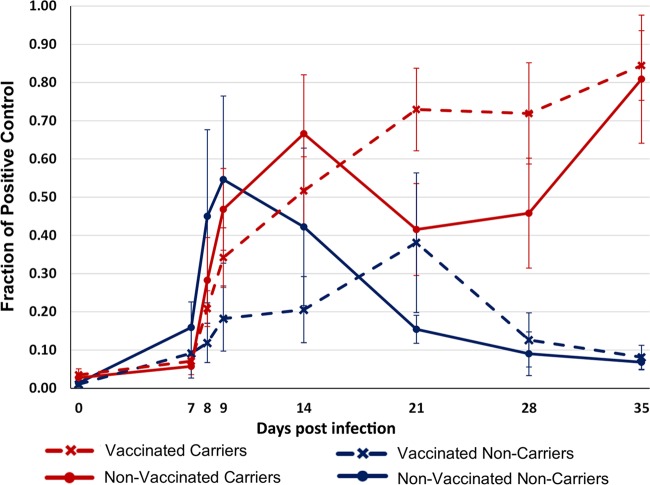
Anti-FMDV IgA levels in saliva measured by an isotype-specific immunosorbent assay. Results are mean values ± SEMs for groups of vaccinated and nonvaccinated cattle further categorized by FMDV persistence status. Data are for 15 vaccinated carriers, 8 nonvaccinated carriers, 4 vaccinated noncarriers, and 5 nonvaccinated noncarriers.

Vaccination by the intramuscular route did not induce detectable levels of anti-FMDV IgA in saliva prior to virus challenge. However, FMDV-specific IgA was detectable in saliva collected from all infected cattle after virus challenge, regardless of vaccination status (Fig. 4). No salivary IgA was detected in the three animals that were presumed sterilely protected. The measurements varied considerably between animals, but by 28 dpi, a substantial difference in the levels of salivary anti-FMDV IgA between carriers and noncarriers was detected, even though the divergence of carrier status was established by 21 dpi (Fig. 4). Beyond this time point, FMDV-specific IgA was detected only in saliva from persistently infected FMDV carriers (Fig. 4).

### Tissue distribution of FMDV. (i) Nonvaccinated cattle, intermediate phase (7 to 14 dpi).

The tissue distribution of FMDV during the preclinical and acute phases of infection, determined using the same virus and infection model used in this study, has been described in a previous publication (30). In the current study, the FMDV distribution during what was defined as the intermediate phase of infection (i.e., before FMDV persistence status could be determined) was investigated in eight nonvaccinated animals, two of which were euthanized on each of 7, 8, 10, and 14 dpi (Table 1). Low levels of FMDV RNA (3.45 log_10_ GCN/ml) but no infectious virus was detected in serum from one animal euthanized at 7 dpi (Table 1, animal 14-46); all other animals in this cohort were postviremic. As a reflection of recent viremia, FMDV RNA detection was close to ubiquitous in tissues collected from nonvaccinated cattle euthanized at 7, 8, and 10 dpi (Table 1). However, the quantities of FMDV RNA and the rates of isolation of infectious virus were variable across anatomic regions. There was an overall trend for the tissue-level detection of infectious virus to decrease through this intermediate phase at all sites with the exception of the sites of virus persistence in the nasopharynx, wherein detection in the intermediate phase was consistent.

**TABLE 1:**
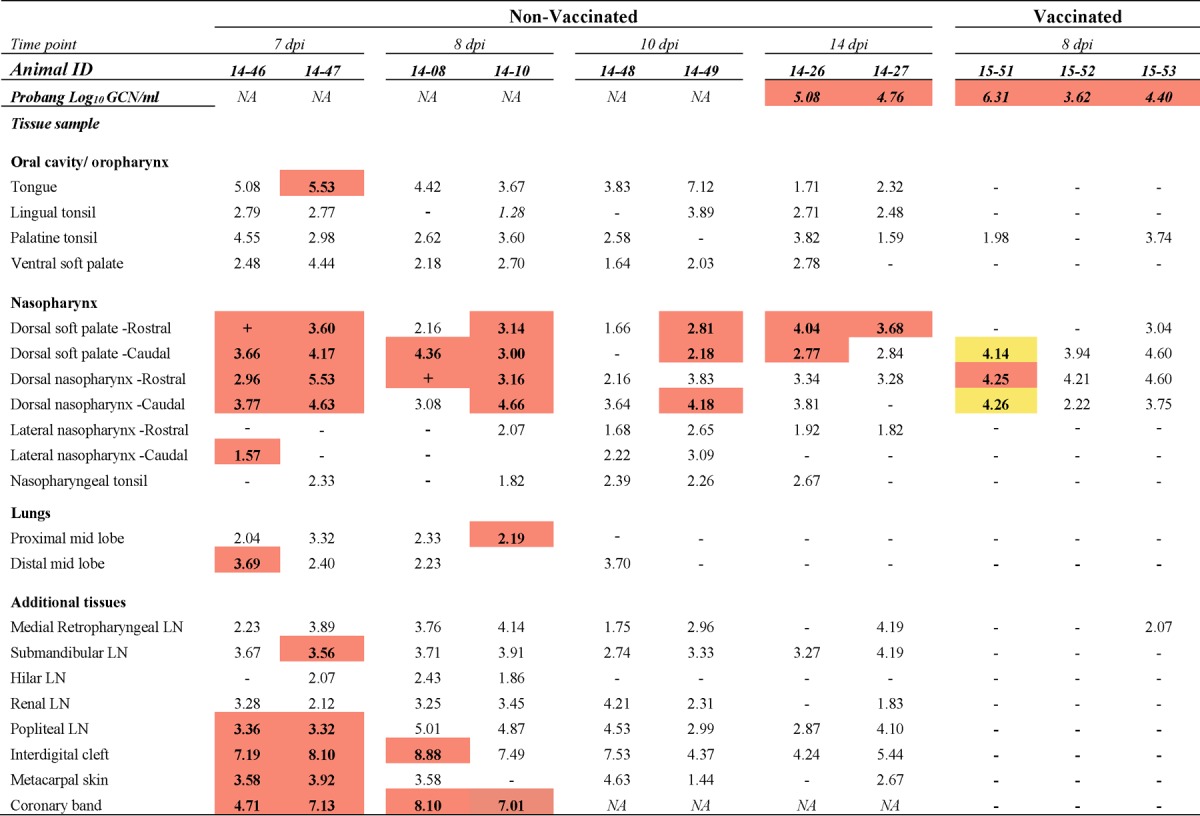
Tissue distribution of FMDV RNA and infectious virus during intermediate phase of infection in nonvaccinated and vaccinated cattle^*a*^

a The numbers in the table represent the log_10_ genome copy number (GCN) per milligram of FMDV RNA in tissue or the log_10_ GCN per milliliter of oropharyngeal fluid (probang samples). Bold numbers in cells with orange shading, the samples were positive for both FMDV RNA (by qRT-PCR) and virus isolation; + in cells with orange shading, virus isolation was positive but the FMDV RNA content was below the limit of detection; −, double-negative samples; bold numbers in cells with yellow shading, the samples were virus isolation positive following TTE treatment of tissue macerates; ID, identifier; NA, not applicable; LN, lymph node. The limits of detection were 1.53 log_10_ FMDV GCN/mg of tissue and 1.57 log_10_ FMDV GCN/ml OPF.

At 7 dpi, FMDV RNA and infectious virus were consistently detected in the dorsal soft palate and dorsal nasopharynx. These distinct mucosal tissues remained the most consistent locations for recovery of infectious FMDV through 8 and 10 dpi, although more variation between animals occurred at these later time points. At 7 and 8 dpi, abundant virus (up to 8.90 log_10_ GCN/mg) was detected in the skin of the interdigital clefts and coronary bands, corresponding to sites of healing vesicular lesions. At 7 dpi, infectious virus was isolated from popliteal and submandibular lymph nodes, which drain lesion predilection sites in the hind feet and oral cavity, respectively. Similarly, FMDV RNA quantities remained comparatively high (up to 5.0 log_10_ GCN/mg) in these lymph nodes at subsequent time points, but infectious virus was not recovered.

FMDV RNA, with sporadic isolation of infectious virus, was present in the lungs of animals euthanized at 7 and 8 dpi and in one animal euthanized at 10 dpi (Table 1). After this point, there was no recovery of viral RNA or infectious virus from pulmonary tissues of any of the animals included in the study. Probang samples harvested prior to euthanasia of the animals at 14 dpi were virus isolation (VI) positive, with virus isolation from tissues being restricted to the dorsal soft palate.

### (ii) Nonvaccinated cattle, persistent phase (≥21 dpi).

Of the 13 nonvaccinated cattle that were kept past the established threshold by which FMDV persistence status could be determined (21 dpi), 8 were persistently infected carriers (defined by detection of infectious virus in probang samples), whereas 5 had cleared the infection (Table 2). FMDV RNA was detected in pharyngeal and/or peripheral tissues, including lesion predilection sites and lymph nodes, of both carriers and noncarriers. However, infectious virus was exclusively isolated from pharyngeal tissues of persistently infected carrier animals that were also shedding infectious virus in probang samples (Table 2). The highest viral loads in tissues (up to 5.60 log_10_ GCN/mg) were detected in nasopharyngeal tissues from which infectious virus could also be isolated. Using the standard VI protocol, there was no detection of infectious virus in tissues of two animals determined to be FMDV carriers on the basis of virus shedding in probang samples. However, infectious FMDV was isolated from the dorsal soft palate of one of these animals (animal 14-107) following the dissociation of immunocomplexed virus by 1,1,2-trichlorotrifluorotethane (TTE) treatment of a replicate tissue sample.

**TABLE 2:**
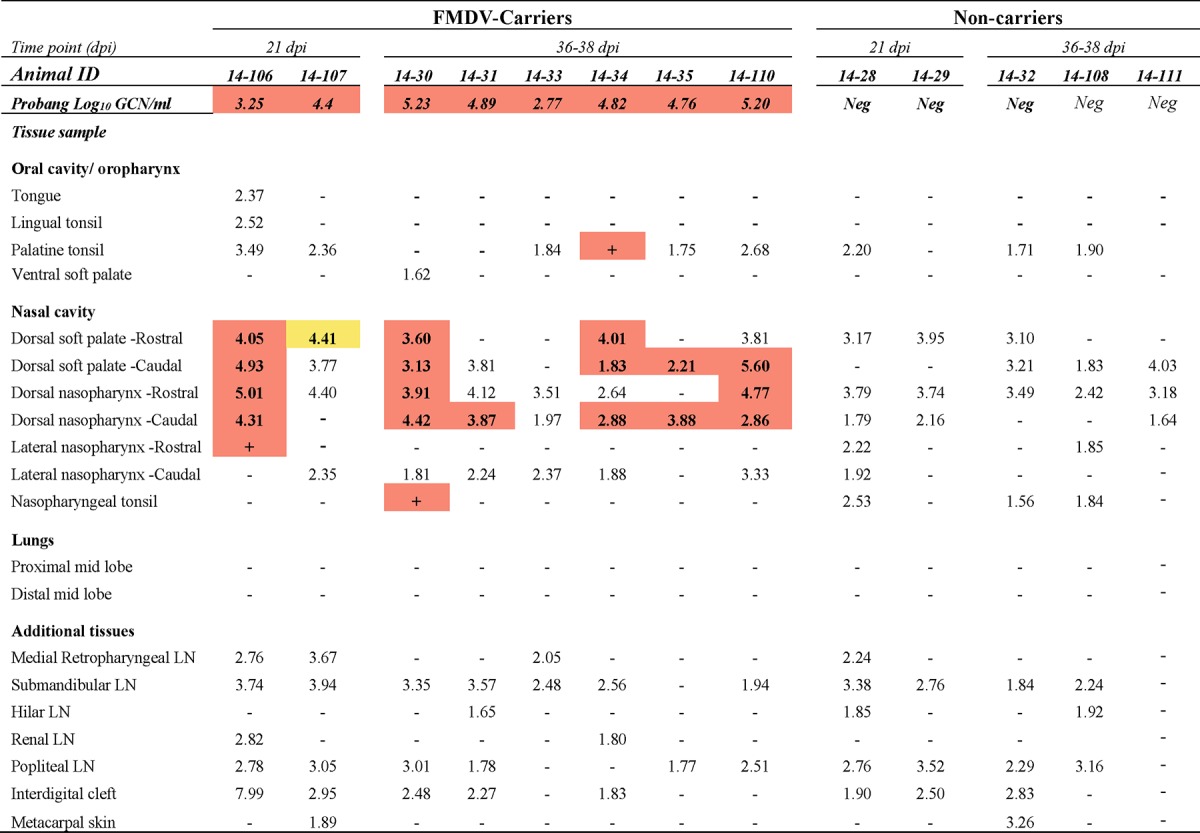
Tissue distribution of FMDV RNA and infectious virus during persistent phase of infection in nonvaccinated cattle^*a*^

a The numbers in the table represent the log_10_ genome copy number (GCN) per milligram of FMDV RNA in tissue or the log_10_ GCN per milliliter of oropharyngeal fluid (probang samples). Bold numbers in cells with orange shading, samples were positive for both FMDV RNA (by qRT-PCR) and virus isolation; + in cells with orange shading, virus isolation was positive but the FMDV RNA content was below the limit of detection; −, double-negative samples; bold numbers in cells with yellow shading, samples were virus isolation positive following TTE treatment of tissue macerates; ID, identifier; Neg, negative; LN, lymph node. The limits of detection were 1.53 log_10_ FMDV GCN/mg of tissue and 1.57 log_10_ FMDV GCN/ml OPF.

### (iii) Vaccinated cattle, intermediate phase (8 dpi).

Three vaccinated cattle were euthanized during the defined intermediate phase of infection (8 dpi; Table 1), before FMDV carrier status could be determined. Probang samples harvested before euthanasia of these animals contained both FMDV RNA and infectious virus. The tissues in which FMDV RNA was detected were restricted to the pharynx, with the largest quantities (up to 4.60 log_10_ GCN/mg) being detected in the dorsal soft palate and dorsal nasopharynx (Table 1). Infectious virus was isolated only from the dorsal soft palate and dorsal nasopharynx from one out of the three cattle (Table 1).

### (iv) Vaccinated cattle, persistent phase (≥10 dpi).

Of the 12 animals that had received the licensed vaccine dose and that survived beyond the threshold by which FMDV carrier status could be determined, 7 were FMDV carriers and 5 were noncarriers (Table 3). Among the 10 animals that received a 10-fold higher vaccine dose, only 2 had cleared the infection, while 8 were persistently infected carriers (Table 4). The localization of infectious virus in tissues of the FMDV carriers of these two vaccinated groups was restricted to the pharynx and was thus comparable to that for the nonvaccinated animals during persistent infection (Tables 2 to 4). Similarly, the FMDV RNA quantities in nasopharyngeal tissues of vaccinated and nonvaccinated cattle were similar. There was, however, a marked difference in the distribution of FMDV RNA in peripheral tissues and lymph nodes between the nonvaccinated animals that had undergone clinical FMD and viremia and the vaccinated animals that had all been protected against clinical disease and dissemination of infection (Tables 2 to 4). Specifically, there was no detection of FMDV RNA or infectious virus at sites distant from the pharynx in any of the vaccinated cattle included in the study. Infectious virus was isolated at a lower prevalence in tissues from cattle that had received the high-dose vaccine than in tissues from animals that had received the standard dose of vaccine, despite the successful isolation of virus from probang samples. This difference was partially abated by TTE treatment of nasopharyngeal tissue samples prior to repeat of VI (Table 4). The VI status of tissues obtained from noncarrier animals did not change following TTE treatment.

**TABLE 3:**
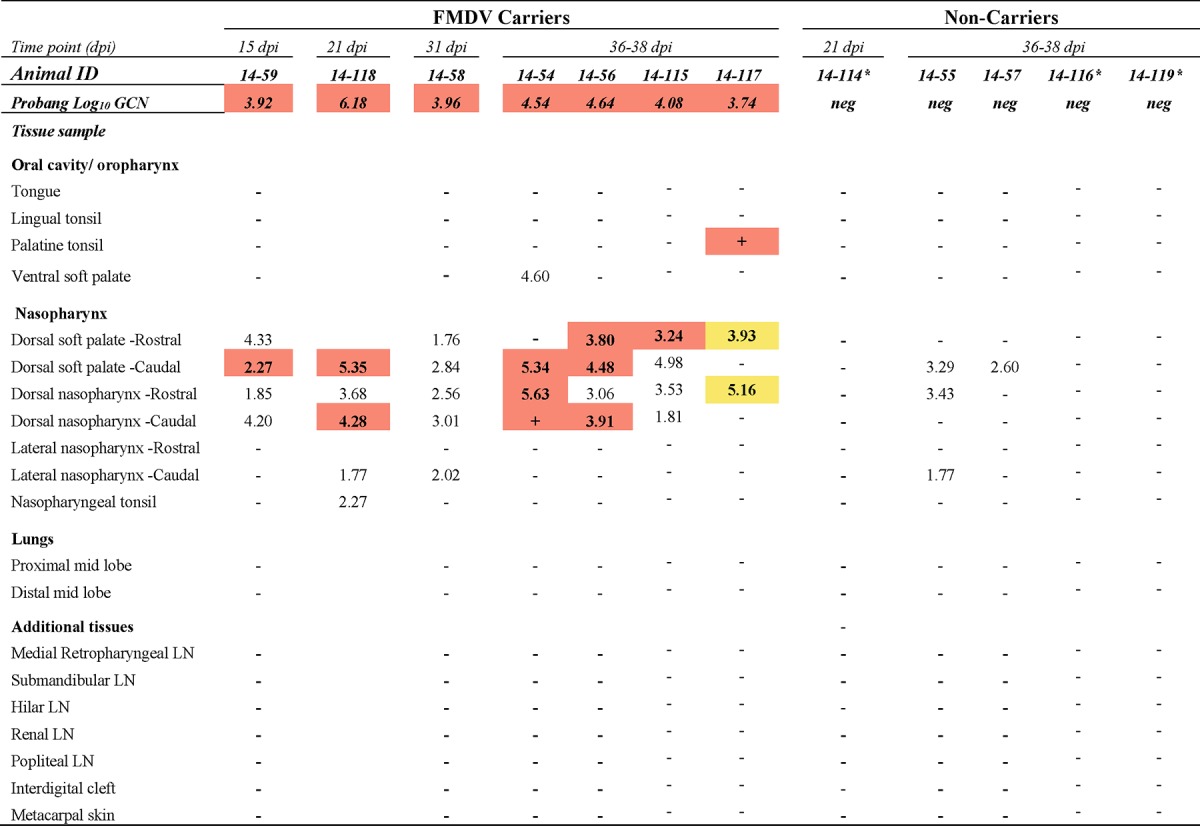
Tissue distribution of FMDV RNA and infectious virus during persistent phase of infection in cattle immunized with licensed vaccine dose^*a*^

a The numbers in the table represent the log_10_ genome copy number (GCN) per milligram of FMDV RNA in tissue or the log_10_ GCN per milliliter oropharyngeal fluid (probang samples). Bold numbers in cells with orange shading, samples were positive for both FMDV RNA (by qRT-PCR) and virus isolation; + in cells with orange shading, virus isolation was positive but the FMDV RNA content was below the limit of detection; −, double-negative samples; bold numbers in cells with yellow shading, samples were virus isolation positive following TTE treatment of tissue macerates; ID, identifier; neg, negative; LN, lymph node; *, presumed sterilely protected cattle. The limits of detection were 1.53 log_10_ FMDV GCN/mg of tissue and 1.57 log_10_ FMDV GCN/ml OPF.

**TABLE 4:**
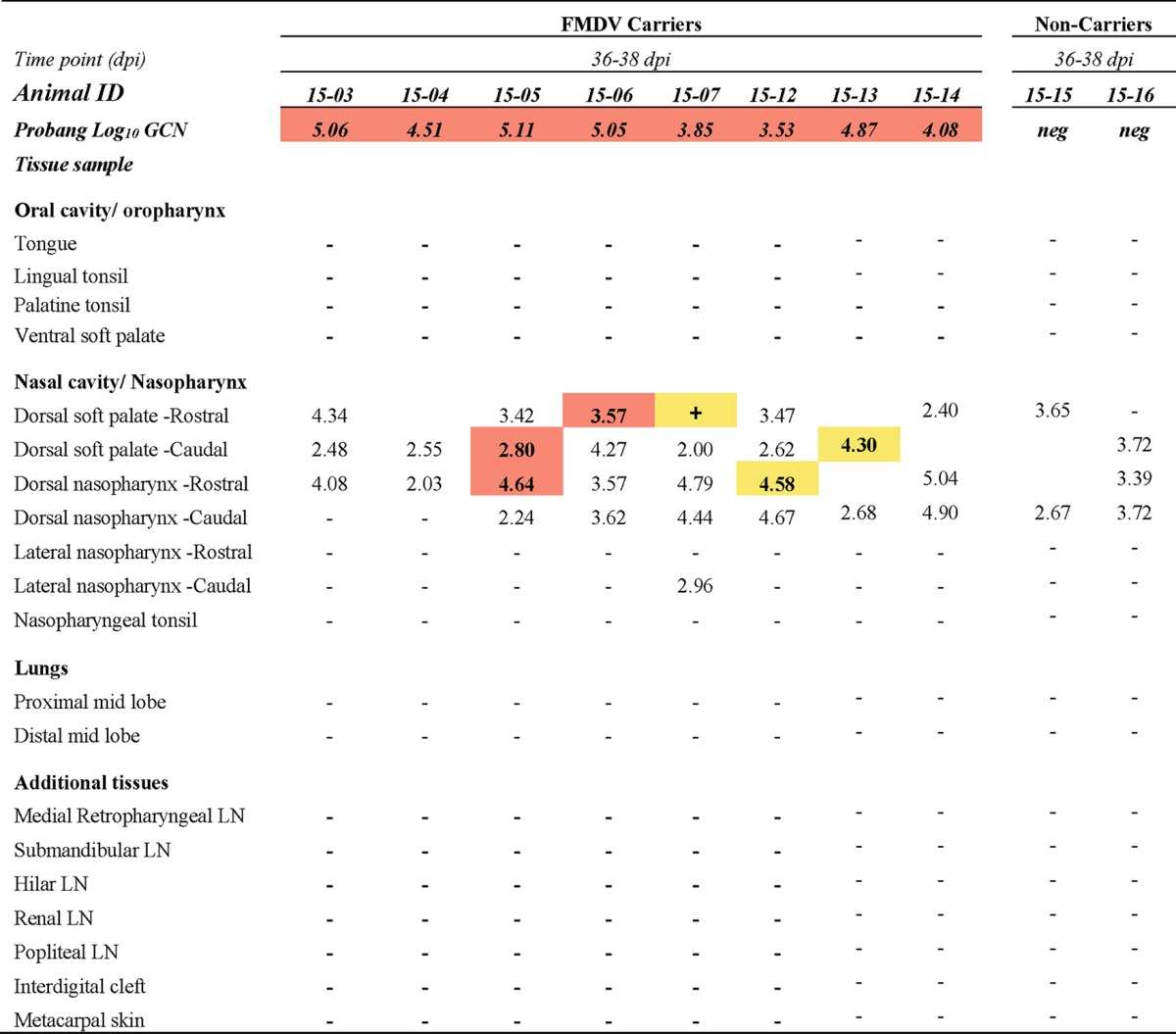
Tissue distribution of FMDV RNA and infectious virus during persistent phase of infection in cattle immunized with 10× licensed vaccine dose^*a*^

a The numbers in the table represent the log_10_ genome copy number (GCN) per milligram of FMDV RNA in tissue or the log_10_ GCN per milliliter oropharyngeal fluid (probang samples). Bold numbers in cells with orange shading, samples were positive for both FMDV RNA (by qRT-PCR) and virus isolation; + in cells with orange shading, virus isolation was positive but the FMDV RNA content was below the limit of detection; −, double-negative samples; bold numbers or symbols in cells with yellow shading, samples were virus isolation positive following TTE treatment of tissue macerates; ID, identifier; neg, negative; LN, lymph node. The limits of detection were 1.53 log_10_ FMDV GCN/mg of tissue and 1.57 log_10_ FMDV GCN/ml OPF.

There was no detection of FMDV RNA or infectious virus in any tissues of three of the five animals in the group receiving the standard dose of vaccine (Table 3, animals 14-114, 14-116, and 14-119). In these three presumed sterilely protected animals, oral and nasal swab specimens collected directly after inoculation contained quantities of FMDV RNA similar to those in the corresponding samples collected from animals with confirmed clinical or subclinical infection (not shown). However, no samples (serum, swab, or tissue samples) harvested from these three animals contained detectable infectious virus or RNA at subsequent time points. In contrast, FMDV RNA was detected in nasopharyngeal tissues of the remaining noncarriers (animals 14-55 and 14-57 [Table 3] and animals 15-15 and 15-16 [Table 4]) of both vaccinated groups, suggesting that these tissues were infected during early infection but that infectious virus had been efficiently cleared.

### Microanatomic distribution of FMDV RNA during persistent infection.

**FIG 5:**
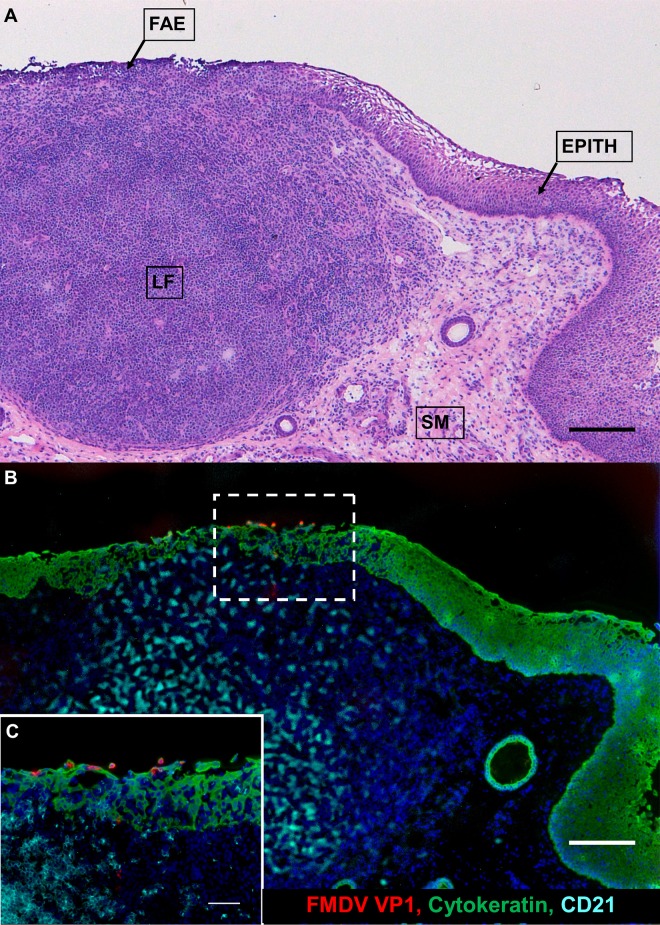
Persistent FMDV infection in bovine nasopharyngeal mucosa. The anatomic localization of persistent FMDV infection in the epithelium of the bovine nasopharyngeal MALT is shown. (A) Dorsal soft palate from a persistently infected FMDV carrier illustrating the histologic characteristics of the specific microanatomic regions involved in persistent FMDV infection. Follicle-associated epithelium (FAE) is superficial to a subepithelial MALT follicle (LF). The FAE is thinner than the adjacent nonlymphoid epithelium (EPITH) with an indistinct basal architecture and contains a heterogeneous population of embedded nonepithelial leukocytes. SM, submucosa. Magnification, ×4; bar, 200 μm. (B) Immunomicroscopic image of a serial section of the image shown in panel A. The FMDV structural protein is predominantly localized to cytokeratin-expressing cells within the FAE. Scarce amounts of FMDV VP1 were also detected within subepithelial lymphoid tissue. Green, cytokeratin; red, FMDV VP1; aqua, CD21; blue, nuclei. Magnification, ×4; bar, 200 μm. (C) Magnification of the region within the hatched rectangle in panel B. Magnification, ×20; bar, 50 μm.

**FIG 6:**
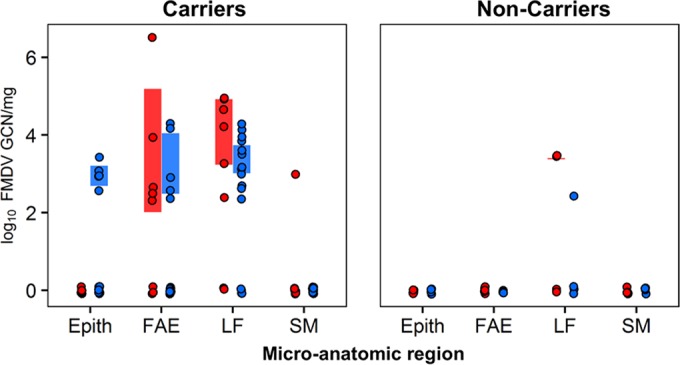
FMDV RNA detection in distinct microanatomic compartments of nasopharyngeal tissue samples derived by LCM. (Left) FMDV genome loads in persistently infected carriers were the highest in follicle-associated epithelium (FAE) and subepithelial MALT follicles (LF). (Right) In noncarriers, FMDV genome detection was scarce and restricted to lymphoid follicles. Red, nonvaccinated animals; blue, vaccinated animals.

Laser capture microdissection (LCM) combined with qRT-PCR was used to determine the FMDV RNA distribution across four distinct microanatomic compartments in nasopharyngeal tissue samples, as has been reported for animals from these same experimental cohorts during the acute phase of infection (30). Viral RNA localization was compared across distinct microanatomic compartments of nasopharyngeal tissue samples (nonlymphoid epithelium [Epith], lymphoid follicle-associated epithelium [FAE], subepithelial lymphoid follicles [LF], and submucosa [SM]) (Fig. 5A), as well as between defined animal categories (nonvaccinated versus vaccinated animals and FMDV carriers versus noncarriers). FMDV RNA was most frequently detected in samples dissected from subepithelial lymphoid follicles and the overlying follicle-associated epithelium of both nonvaccinated and vaccinated carriers (Fig. 6A). Viral RNA was detected less frequently in the nonlymphoid epithelium and submucosa from nonvaccinated and vaccinated FMDV carriers, whereas FMDV RNA detection in LCM samples from noncarriers was scarce and restricted to subepithelial lymphoid follicles (Fig. 6B).

### Quantitation of cytokine mRNA in microdissected nasopharyngeal tissues.

**FIG 7:**
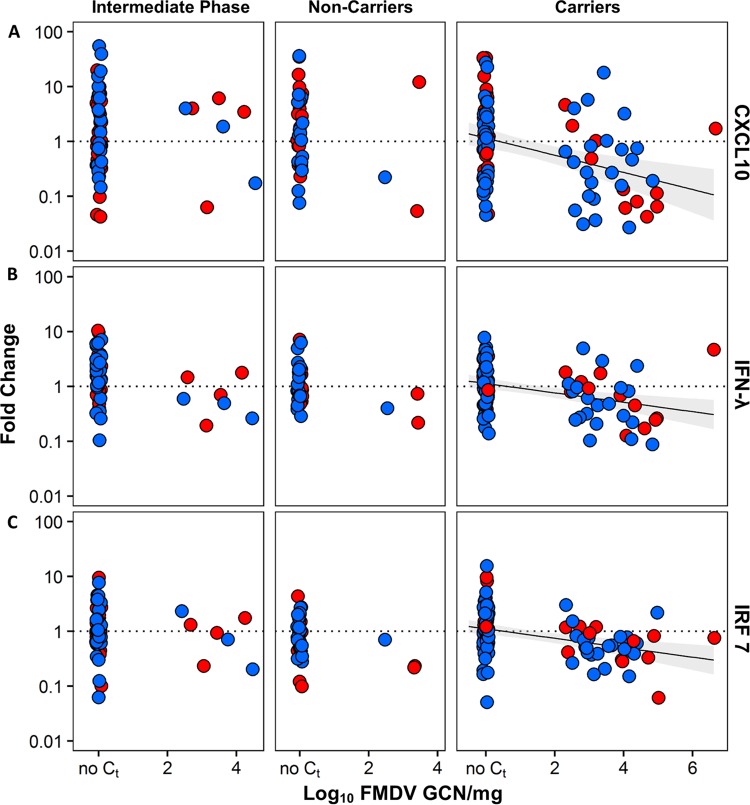
Expression of CXCL10 (A), IFN-γ (B), and IRF-7 (C) mRNA plotted against FMDV genome load. Combined linear regression lines for nonvaccinated animals (red) and vaccinated animals (blue) are shown with 95% confidence intervals (areas shaded gray). In persistently infected carriers, CXCL10, IFN-γ, and IRF-7 mRNA expression levels were negatively correlated with the FMDV genome load.

The relative expression (normalized to that of the host housekeeping gene GAPDH and compared to that for uninfected animals) of IFN-λ, IFN-γ, IRF-7, CXCL10, and IL-10 mRNA in total RNA extracted from LCM-derived nasopharyngeal tissues of carriers and noncarriers was quantified (Fig. 6 and 7). Host gene expression was compared using the same experimental design and the same RNA samples used to compare the viral RNA distribution. There was no significant association between vaccination status and host gene expression. Thus, data from vaccinated and nonvaccinated cattle were pooled and stratified by FMDV carrier status for analysis of gene expression. Cattle euthanized during the intermediate phase of infection (before their carrier status was determined) were treated as a separate group in the data analysis.

Analysis across all microdissected tissues indicated that the regional expression of all evaluated host gene mRNAs (mRNAs for cytokines and transcription factors) was negatively correlated with the presence and level of detection of viral RNA (Table 5; Fig. 7). This correlation was significant for all genes examined (Holm's method-adjusted *P* values, <0.05). The slopes of all regression lines were negative, with the steepest slope being estimated for the CXCL10-FMDV GCN regression, followed by the IFN-γ–FMDV GCN and IRF-7–FMDV GCN regressions. None of the confidence intervals for the slopes of the curves included 0, providing further evidence for a negative association between FMDV RNA quantities and expression of the evaluated host mRNAs.

**TABLE 5:** Correlation between expression levels of host gene mRNA and between FMDV GCN and host gene mRNA expression in LCM-processed nasopharyngeal tissue samples^*a*^

Host gene	Correlation with FMDV GCN			Correlation (*r*) between host gene expression levels				
	Slope β (95% CI)	*r*	*P* value	IFN-γ	IFN-λ	IL-10	CXCL10	IRF7
IFN-γ	−0.36 (−0.49 to −0.23)	**−0.35**	**<0.001**	1	**0.36**	**0.47**	**0.58**	**0.50**
IFN-λ	−0.26 (−0.43 to −0.09)	**−0.21**	**0.016**	**0.36**	1	**0.49**	0.17	−0.01
IL-10	−0.21 (−0.32 to −0.11)	**−0.28**	**0.001**	**0.47**	**0.49**	1	0.21	**0.43**
CXCL10	−0.50 (−0.73 to −0.27)	**−0.31**	**<0.001**	**0.58**	0.17	0.21	1	0.31
IRF7	−0.26 (−0.38 to −0.14)	**−0.28**	**0.001**	**0.50**	−0.01	**0.43**	0.31	1

a Bold and underscored values indicate a statistically significant correlation between variables (*P* < 0.05).

There were significant positive correlations (Holm's method-adjusted *P* values, <0.05) between the levels of IFN-γ mRNA expression and the levels of expression of all other genes (mean *r* = 0.48), as well as between the levels of expression of IFN-λ and IL-10 mRNA and the levels of expression of IRF-7 and IL-10 mRNA (Table 5). Thus, within microdissected nasopharyngeal tissues of carrier-phase cattle, upregulation of IFN-γ mRNA occurred with concurrent induction of IFN-λ, IL-10, IRF-7, and CXCL10. Similarly, there was a significant association between the induction of IL-10 mRNA and that of IFN-λ and IRF-7 mRNA.

**FIG 8:**
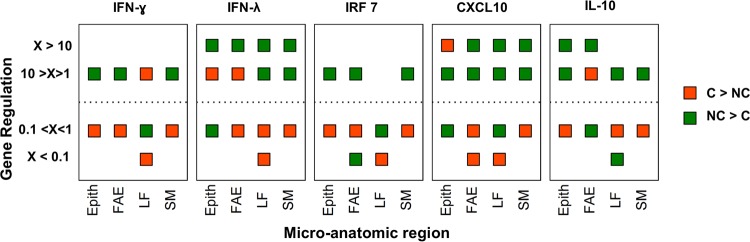
Host factor modulation in nasopharyngeal tissues from FMDV carriers (C) and noncarriers (NC). Host factor mRNA measurements in LCM-derived samples of nasopharyngeal tissues were assigned to four strata, separating observations with greater than 10-fold up- or-downregulation from observations with less up- or downregulation. The color of the data points indicates if the proportion of up- or downregulation for each host factor and microanatomical region within the defined strata was higher in carriers (orange) or noncarriers (green). Noncarriers had a more consistent induction of the targets measured.

Additionally, a semiquantitative analysis was performed to evaluate potential differences in the relative frequencies of up- or downregulation of the measured host factor mRNAs in microdissected pharyngeal tissues between carriers and noncarriers. The modulation of gene expression was stratified to differentiate fold changes (positive or negative) of greater than 10 from fold changes of between 1 and 10. There was a higher frequency of relative gene upregulation among noncarriers than carriers (Fig. 8). Specifically, a greater than 10-fold upregulation of IFN-λ, IL-10, and CXCL10 mRNAs occurred more frequently in noncarriers. Similarly, there were more events of relative downregulation of host gene expression among FMDV carriers (Fig. 8).

**FIG 9:**
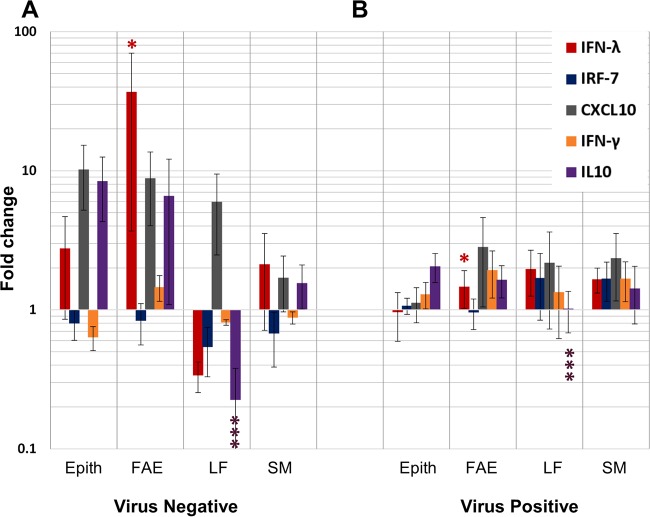
Host factor mRNA expression in microdissected samples of nasopharyngeal mucosa harvested during the transitional phase of infection in nonvaccinated cattle. IFN-λ, IRF-7, CXCL10, IFN-γ, and IL-10 mRNA expression levels in microdissected tissue compartments of nasopharyngeal mucosa from FMDV-negative (A) (*n =* 3) and FMDV-positive (B) (*n =* 5) cattle euthanized at 10, 14 or 21 dpi, corresponding to the period of divergence to carrier versus noncarrier status, were measured by qRT-PCR and are reported as the mean fold change ± SEM within each animal category. The expression levels are normalized to those of a housekeeping gene (GAPDH) and are relative to baseline levels established from similar analyses of tissues from noninfected cattle. Animals that had recently cleared the infection (A) (virus negative) had significantly higher levels of IFN-λ mRNA expression in follicle-associated epithelium (FAE) (*, Holm's method-adjusted *P* value, ≤0.05) and significantly lower levels of IL-10 mRNA expression in subepithelial lymphoid follicles (LF) (***, Holm's method-adjusted *P* value, ≤0.001) than virus-positive animals.

In order to evaluate possible associations between host factor mRNA expression levels and the processes of clearance of FMDV from tissues, a separate analysis of relative gene expression was performed by including samples from nonvaccinated animals euthanized between 10 and 21 dpi, representing the time frame of divergence (transitional phase). As most of these animals lacked a defined FMDV persistence status, they were grouped on the basis of detection of infectious virus in tissues or probang samples and thus categorized as either virus positive (presumptive carriers, *n =* 5) or virus negative (presumptive noncarriers, *n =* 3). Among these animals, there was a significantly higher level of IFN-λ mRNA expression in the follicle-associated epithelium of FMDV-negative cattle, corresponding to the individuals believed to have recently cleared the infection (Holm's method-adjusted *P* value, ≤0.05; Fig. 9). In contrast, in the same FMDV-negative cattle, the level of IL-10 mRNA expression within mucosa-associated lymphoid follicles was significantly lower than that in animals that were still carrying the infection (Holm's method-adjusted *P* value, ≤0.001; Fig. 9). Additionally, there was a trend, though it was not statistically significant, of higher relative levels of expression of IL-10 and CXCL10 mRNAs in the nonlymphoid epithelium and follicle-associated epithelium of cattle that had cleared the infection (Fig. 9).

### Localization of FMDV antigen by immunomicroscopy.

**FIG 10:**
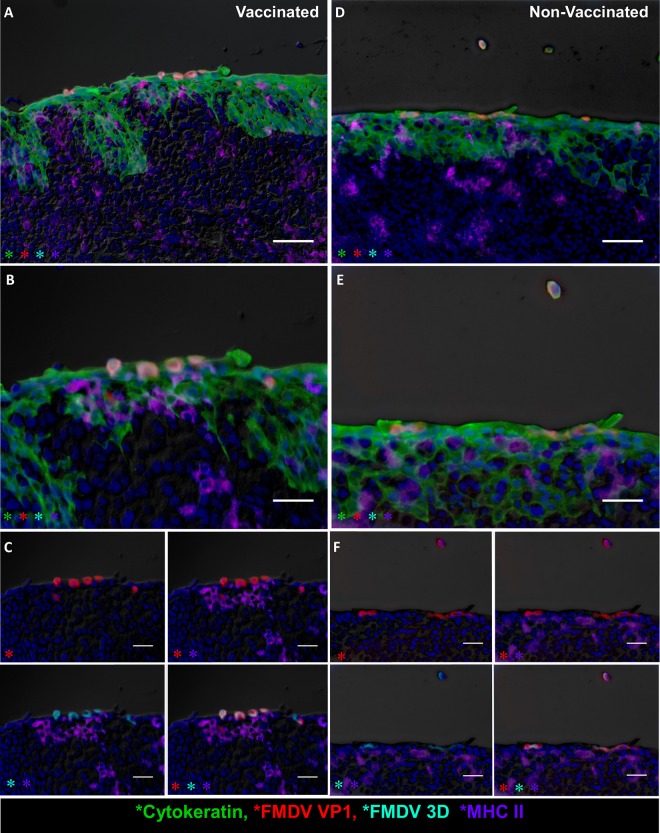
FMDV structural and nonstructural proteins within follicle-associated epithelium of the bovine nasopharyngeal mucosa. The microscopic distributions of FMDV structural (VP1) and nonstructural (3D) proteins in the dorsal soft palate of vaccinated (A to C) and nonvaccinated (D to F) cattle at 35 dpi were determined by the multichannel immunofluorescence technique. (A) Dorsal soft palate of a vaccinated steer at 35 dpi. FMDV VP1 (red) and 3D (aqua) proteins colocalize with cytokeratin (green) in foci of persistent FMDV infection in the superficial layer of follicle-associated epithelium. MHC-II^+^ cells (purple) are present in the subepithelium. Magnification, ×20; bar, 50 μm. (B) Magnification of the region of interest in panel A. Magnification, ×40. (C) Select channel combinations demonstrating colocalization of FMDV VP1 (red) and FMDV 3D (aqua) with cytokeratin (green) but not with MHC-II (purple). Bars, 25 μm. (D) Dorsal soft palate of a nonvaccinated steer at 35 dpi. FMDV VP1 (red) and 3D (aqua) proteins colocalize with cytokeratin (green) within follicle-associated epithelium. Intraepithelial MHC-II^+^ cells (purple) are in close proximity to virus-infected epithelial cells. Two VP1-, 3D-, and cytokeratin-positive cells are sloughing off the epithelial surface. Magnifications, ×20; bar, 50 μm. (E) Magnification of the region of interest in panel D. Magnification, ×40. (F) Select channel combinations demonstrating FMDV VP1 (red) and 3D (aqua) colocalization with cytokeratin-expressing cells (green) but not with MHC-II-expressing cells (purple). Bars, 25 μm.

**FIG 11:**
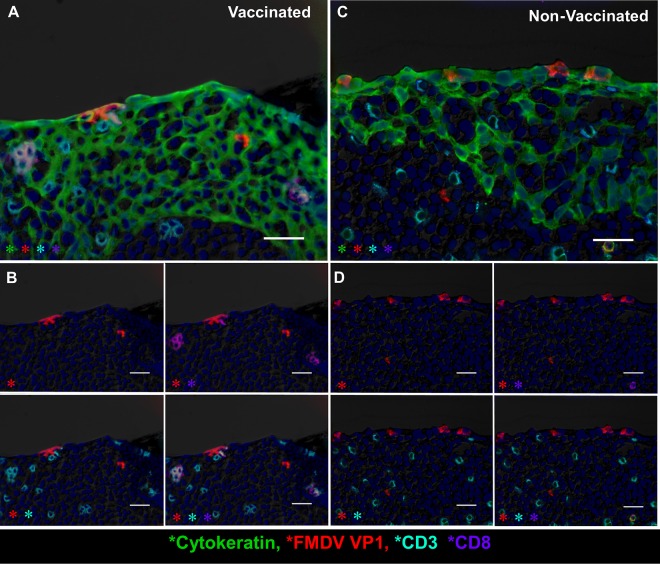
Variable T-cell populations in close proximity of foci of persistent FMDV infection in bovine nasopharyngeal mucosa determined by the multichannel immunofluorescence technique. (A) FMDV VP1 (red) protein within cytokeratin-expressing epithelial cells (green) in the follicle-associated epithelium of the dorsal soft palate of a vaccinated steer at 35 dpi. CD8^+^ (purple)/CD3^+^ (aqua) double-positive cytotoxic T lymphocytes and CD8^−^/CD3^+^ (presumptive T-helper) lymphocytes are present in the subepithelial compartment and interspersed within the epithelium. Magnification, ×40; bar, 25 μm. (B) Select channels of the image shown in panel A. CD3^+^ cells (aqua) include single-positive (T-helper cells) or CD8^+^/CD3^+^ double-positive cytotoxic T lymphocytes. Additional individual cells of a CD3^−^/CD8^+^ phenotype represent presumptive NK cells. Bars, 25 μm. (C) The FMDV VP1 (red) protein colocalizes with cytokeratin (green) in lymphoid follicle-associated epithelium of the dorsal soft palate of a nonvaccinated steer at 35 dpi. T-helper cells (CD3^+^/CD8^−^) and cytotoxic T lymphocytes (CD3^+^/CD8^+^) are located directly adjacent to FMDV-infected cells in the superficial layer of the epithelium. Magnification, ×40; bar, 25 μm. (D) Individual channels of the image shown in panel C showing CD3 (aqua) single-positive or CD3/CD8 (aqua/purple) double-positive T lymphocytes in direct proximity to FMDV VP1 (red). Magnifications, ×40; bars, 25 μm.

In order to characterize the microscopic virus distribution and host cellular responses to infection, pharyngeal tissues from experimentally infected cattle were examined by multichannel immunofluorescence microscopy (MIF). FMDV structural (VP1) and nonstructural (3D) proteins were exclusively detected in nasopharyngeal tissue samples obtained from persistently infected carriers. The most consistent localization of viral protein was within the follicle-associated epithelium of the dorsal nasopharynx or dorsal soft palate (Fig. 5B and C, 10, and 11). FMDV VP1 and 3D colocalized with cytokeratin in single cells or small clusters of a few adjacent cells within superficial layers of this microanatomically distinct compartment (Fig. 10 and 11). MHC-II-expressing (MHC-II^+^) cells were detected close to FMDV-infected epithelial foci but did not colocalize with viral proteins (Fig. 10). Additionally, CD3^+^ and CD3^+^/CD8^+^ cells representing presumptive T-helper cells, γδ-T cells, or cytotoxic T lymphocytes were detected directly adjacent to FMDV-infected epithelial cells, but without direct colocalization (Fig. 11).

**FIG 12:**
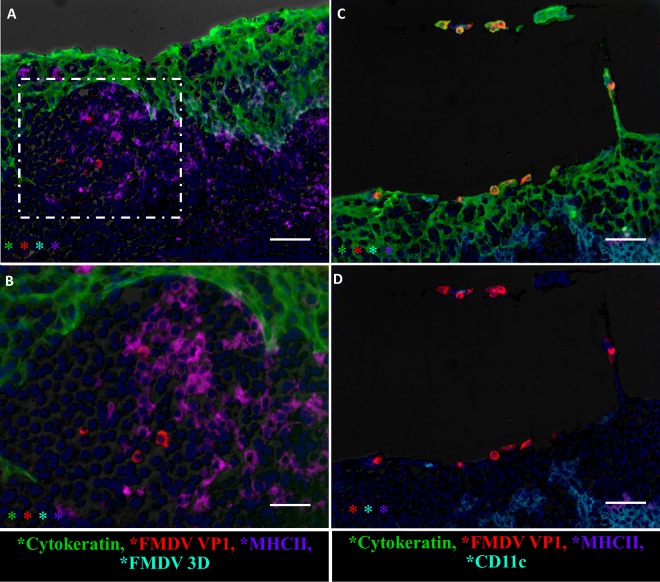
Rare events in localization of FMDV during persistent infection determined by the multichannel immunofluorescence technique. (A) FMDV VP1 (red) localization in subepithelial lymphoid tissue of the dorsal soft palate of a vaccinated steer at 35 dpi. Magnification, ×20; bar, 50 μm. (B) Higher magnification of the region within the hatched rectangle in panel A. FMDV VP1 (red) is in close association and partially colocalized with MHC-II (purple) in lymphoid tissue subjacent to the follicle-associated epithelium (green). There was no concurrent detection of the FMDV 3D antigen (aqua; no signal detection). Magnification, ×40; bar, 25 μm. (C) FMDV-infected (red), cytokeratin-positive (green) epithelial cells sloughing off from the area of lymphoid follicle-associated epithelium of the dorsal soft palate of a vaccinated steer at 35 dpi. CD11c-expressing (aqua) antigen-presenting cells and CD8^+^ (purple) lymphocytes are present below and within the epithelial compartment but are not colocalized with virus. (D) Three-channel combination of the image shown in panel C.

In addition to epithelial localization, there was rare detection of the FMDV structural (VP1) protein within subepithelial mucosa-associated lymphoid tissue (MALT) follicles (Fig. 5B and C, 11, and 12). Within the subepithelial compartment, FMDV VP1 was detected as clusters of a few virus-positive cells in close association with MHC-II^+^ cells, but without concurrent detection of FMDV 3D (Fig. 12). On rare occasions, sloughing and separation of virus-infected epithelial cells (VP1^+^ and cytokeratin expressing) from nasopharyngeal mucosal surfaces were detected (Fig. 12). The microanatomic distribution of FMDV antigen, as well as the detection frequency and phenotypic characteristics of associated cells, was similar between nonvaccinated and vaccinated FMDV carriers.

## DISCUSSION

The existence of a prolonged, subclinical phase of FMDV infection in cattle was first described by van Bekkum et al. (23), with further characterization through experimental studies being conducted by Sutmoller and Gaggero (7). Since then, multiple works have confirmed that the FMDV carrier state occurs in both nonvaccinated and vaccinated cattle, regardless of preoccurring clinical FMD and despite the presence of a marked humoral immune response (21, 26, 33, 55–59; M. Eschbaumer and C. Stenfeldt, unpublished data). Nonetheless, the detailed mechanisms underlying the FMDV carrier state, including the host-virus interactions that facilitate long-term persistence of infectious virus, have not been thoroughly elucidated. The current work is the product of a series of experimental studies dedicated to an overarching goal of elucidating the subclinical divergence that defines whether FMDV-infected cattle succeed in complete clearance of virus or maintain persistent infection.

Detailed monitoring of the dynamics of virus shedding in oropharyngeal fluid (probang samples) confirmed that it was possible to determine the FMDV persistence status of vaccinated animals at 10 dpi. Furthermore, the current investigations found that divergence in nonvaccinated cattle consistently occurred by 21 dpi. This is in contrast to the commonly accepted threshold that defines FMDV persistence on the basis of virus detection beyond 28 dpi. However, earlier studies investigating the persistence of FMDV serotype O have similarly suggested that the divergence between FMDV carriers and noncarriers occurred by 14 dpi (24). Consistent with previous works (58), the current study found that the quantities of FMDV RNA shed in oral and nasal secretions were significantly reduced by vaccination. However, moderately increased amounts of FMDV RNA were detected in probang samples from vaccinated cattle. Additionally, the current study confirmed previous findings (60) which demonstrated that swab samples are inadequate for detection of FMDV during the persistent phase of infection.

The anatomic localization of infectious virus was consistent with that described in previous works and confirmed that the nasopharyngeal mucosa is the site of persistent FMDV (21, 25, 26). Furthermore, a time course of sequential postmortem sampling of nonvaccinated animals demonstrated a gradual clearance of virus from peripheral sites during the transitional phase of infection, with selective persistence of infectious virus in the nasopharynx being seen in a subset of animals. The localization of infectious virus during persistent infection was similar in nonvaccinated and vaccinated cattle. Interestingly, the anatomic distribution of FMDV during persistent infection closely resembled the virus localization during early, previremic infection (27–30). This further reinforces the concept that the bovine nasopharyngeal epithelium has unique tropism-defining properties which have still not been fully elucidated.

In contrast to the similar distribution of infectious virus in vaccinated and nonvaccinated cattle during the persistent phase of infection, the detection of residual FMDV genomic RNA was markedly different between treatment groups. The distribution of FMDV RNA in the tissues of nonvaccinated cattle during late infection resembled the wide dissemination of virus that had occurred during viremia and clinical FMD. In contrast, there was no detection of the FMDV genome at sites distant from the pharynx in the vaccinated group, corresponding to the overall highly restricted distribution of virus (and a lack of systemic dissemination) in these animals. Furthermore, within either the nonvaccinated or the vaccinated cohorts, the anatomic distribution of the FMDV genome was largely similar between carriers and noncarriers.

The detection of residual FMDV RNA in tissues from animals that had cleared the infection is consistent with the findings of previous investigations in cattle and pigs (12, 26). The strong correlation between the tissue distribution of FMDV RNA during the persistent phase of infection and the dissemination of virus during early infection suggests that this persistent FMDV RNA represents residual viral remnants in tissues previously exposed to infectious virus. This is in striking contrast to the distribution of persistent virus, which was exclusively localized to the nasopharyngeal mucosa, regardless of the previous systemic dissemination of virus. Thus, the presence of infectious virus in oropharyngeal fluid and pharyngeal tissues defined the distinction between carriers and noncarriers, whereas residual FMDV RNA could be detected in the tissues of all previously infected cattle, regardless of carrier status. It was not possible to isolate infectious FMDV from tissues by conventional VI from a subset of cattle from which probang samples were consistently VI positive. This inability to isolate virus from tissues obtained from animals defined to be carriers by VI from probang samples was most common among cattle that had received a 10-fold increased vaccine dose, which is consistent with confirmed higher levels of anti-FMDV IgG and IgM in serum at the time of necropsy of the cattle in this group (data not shown). Furthermore, detection of virus in tissues by VI was improved by pretreating tissue macerates with TTE, which is believed to facilitate the dissociation of immunoglobulin-virus complexes (26, 37). Overall, the combined output from these findings suggests that sequential probang sampling is a more sensitive tool for the detection of persistent FMDV infection in cattle than necropsy and postmortem tissue collection and analysis. Additionally, under the conditions of these studies, it is apparent that detection of infectious FMDV from tissues is impeded by anti-FMDV immunoglobulin.

Three vaccinated steers from the current investigation were distinguished from all other animals included in the study on the basis of a lack of any evidence of infection subsequent to virus challenge (presumed sterile immunity). This determination included a lack of detection of FMDV RNA or infectious virus in any sample obtained beyond 24 h postinoculation (including swab, probang, and tissue samples) and a lack of seroconversion to the presence of antibodies against FMDV nonstructural proteins (data not shown). With the exception of these three individuals, FMDV RNA was present in oral and nasal secretions and tissues from all cattle, regardless of whether the animals developed persistent FMDV infection or not. It cannot be determined if the absence of infection in these three individuals was due to intrinsic host factors or vaccine-induced protection. However, further characterization of the host processes associated with this resistance to FMDV infection could potentially elucidate novel aspects of protective immunity.

Immunomicroscopic analyses consistently confirmed the localization of replicating FMDV to specific regions of the nasopharyngeal mucosa (the follicle-associated epithelium of the dorsal soft palate and/or dorsal nasopharynx). FMDV structural (VP1) and nonstructural (3D) proteins were localized to scarce clusters of cytokeratin-positive epithelial cells within the superficial layer of these distinct segments of epithelium. In contrast to observations during the early phases of infection (27, 30), there were no signs of erosions or marked infiltration of antigen-presenting cells associated with foci of persistent FMDV. This suggests that unlike acute infection, during the carrier state, FMDV infection of the pharynx is noncytolytic and immunologically sequestered.

Additional immunomicroscopy imaging demonstrated FMDV-infected epithelial cells sloughing from the nasopharyngeal mucosal of persistently infected cattle. These findings confirm that virus shed during the persistent phase of FMDV infection is largely cell associated and further explain why the use of a probang sampling cup is a superior method for the successful harvest of virus samples from FMDV carriers.

A previous investigation by Juleff et al. (31) postulated a mechanism of FMDV persistence through retention of viable virus particles by the follicular dendritic cell (FDC) network of lymphoid tissue draining the oral cavity. In the current study, FMDV RNA was detectable in the submandibular lymph nodes of the majority of cattle that had undergone clinical FMD. However, despite TTE treatment of tissue macerates, it was not possible to isolate infectious FMDV from any lymph nodes obtained after resolution of the clinical phase of disease. Despite the lack of detection of infectious virus from lymphoid tissue, localization of FMDV RNA and structural antigen to subepithelial lymphoid (MALT) follicles was confirmed by LCM and immunomicroscopy. There was, however, no detection of the FMDV nonstructural protein in these regions. Thus, the findings of the current study do not provide any evidence of actively replicating virus in lymphoid tissues during FMDV persistence. The precise mechanisms involved in the localization of FMDV RNA and capsid antigen to lymphoid follicles are not known. It is possible that accumulation of FMDV degradation products in subepithelial tissue may represent the transport of FMDV from the epithelium to subjacent lymphoid tissues. However, further investigations are required to elucidate the possible relevance of detection of nonreplicating viral components in lymphoid tissue during persistent FMDV infection.

The application of LCM enabled microscopic compartmentalization and segregation of nasopharyngeal tissue samples for downstream analyses of viral and host RNAs. This technique was particularly important in the current study because of the anatomic heterogeneity of the bovine nasopharynx and the highly restricted, focal distribution of FMDV to distinct epithelial segments. Thus, microanatomic dissection of tissue compartments prior to qRT-PCR analysis ensured the homogeneity of the analyzed tissue and facilitated the potential differentiation of virus distribution and host gene expression in specific anatomic and physiological compartments. Using this approach, quantification of the expression levels of select antiviral host factor mRNAs within distinct microanatomic tissue compartments indicated significant negative correlations between FMDV genome loads and antiviral activity. Thus, in the pharyngeal tissues of carrier cattle, the presence of FMDV RNA was associated with the inhibition of several host antiviral genes. This finding is in contrast to the findings of some previous works which demonstrated the induction of other proinflammatory cytokines during the FMDV carrier state (53, 61).

The negative correlation was most pronounced for CXCL10 (IFN-γ-inducible protein 10 [IP-10]), IFN-γ, and IRF-7 mRNAs. CXCL10 is induced by IFN-γ and functions as a chemoattractant for activated T cells. This chemokine is secreted upon stimulation by type I and II interferons, and a dysfunctional or abolished CXCL10 response has previously been associated with failure of the clearance of virus infections (62, 63). IRF-7 is a transcription factor with a central function in the activation pathway of virally induced IFN production (64). Previous works from our laboratory have demonstrated the induction of CXCL10 and IRF-7 in bovine tissues during acute FMDV infection (46), while they have suggested the downregulation of CXCL10 during FMDV persistence (26). Interestingly, the protection of cattle against acute FMDV infection conferred by exogenously administered bovine IFN-λ has been associated with the upregulation of both CXCL10 and IRF-7 in tissues and peripheral blood mononuclear cells (65, 66). Additionally, a high level of expression of IFN-γ has been associated with the inhibition of FMDV replication and the clearance of persistent infection in tissue culture (67).

In further support of the presumed underexpression of antiviral host factors associated with FMDV persistence, the current study demonstrated an overexpression of IFN-λ mRNA in lymphoid follicle-associated epithelium of transitional-phase cattle that had recently cleared the infection. Although the causality and functional mechanisms of induction or suppression of antiviral factors are not completely elucidated herein, the significant association between FMDV persistence and the disruption of select antiviral pathways may be indicative of the failure of local antiviral immunity and, thus, a lack of viral clearance.

In conclusion, the work presented herein provides the most detailed investigation of the mechanisms of FMDV persistence in cattle performed to date. Under the conditions of the current study, the subclinical divergence dictating the persistence versus clearance of virus occurred earlier than the recognized definition of 28 dpi (10 dpi for vaccinated cattle or 21 dpi for nonvaccinated cattle). Our findings surpass those of previous works which localized the anatomic site of FMDV persistence to the bovine nasopharynx by demonstrating the localization of FMDV structural and nonstructural proteins to pharyngeal epithelial cells in vaccinated and nonvaccinated cattle. Additionally, we have demonstrated the downregulation of distinct antiviral host factors within microanatomic compartments containing persistent virus, which may be directly related to the carriers' failure to clear the infection. Further investigations of greater breadth, including expanded transcriptome analyses and manipulation of host factors associated with FMDV persistence, are ongoing in our laboratory.
